# Lymphocyte and NK Cell Counts Can Predict Sepsis-Associated Delirium in Elderly Patients

**DOI:** 10.3389/fnagi.2020.621298

**Published:** 2021-01-11

**Authors:** Dongkai Li, Jiahui Zhang, Guangxu Bai, Jianwei Chen, Wei Cheng, Na Cui

**Affiliations:** Department of Critical Care Medicine, Peking Union Medical College Hospital, Beijing, China

**Keywords:** delirium, sepsis-associated delirium, lymphocyte subsets, NK cell, elderly people

## Abstract

**Background**: Sepsis-associated delirium (SAD) is prevalent in elderly patients and is recognized as brain dysfunction associated with increased inflammatory response in the central nervous system during sepsis. Neuroinflammation was demonstrated to be part of its mechanism and we aimed to validate the role of immunity imbalance in a combined retrospective and prospective cohort study.

**Methods**: We performed a retrospective study analyzing the association between SAD and lymphocyte counts in the peripheral blood, alongside a prospective trial evaluating the quantitative changes in lymphocyte subsets and their predictive value for early diagnosis of SAD.

**Results**: In the retrospective study, among 1,010 enrolled adult patients (age ≥65 years), 297 patients were diagnosed with delirium during intensive care unit (ICU) stay and lymphocyte counts at ICU admission in the SAD group were significantly higher than in non-delirious counterparts (1.09 ± 0.32 vs. 0.82 ± 0.24, respectively, *p* = 0.001). In the prospective study, lymphocyte counts [0.83 (0.56, 1.15) vs. 0.72 (0.40, 1.06) × 10^9^/L, *p* = 0.020] and natural killer (NK) cell counts [96 (68, 118) vs. 56 (26, 92) cells/μl, *p* = 0.024] were significantly higher in the SAD group. The area under the curve value of NK cell count was 0.895 [95% confidence interval (CI): 0.857, 0.933] and of lymphocyte count was 0.728 (95% CI: 0.662, 0.795). An NK cell count cut-off value of 87 cells/ml in septic patients at ICU admission was predictive of delirium with a sensitivity of 80.2% and specificity of 80.8%.

**Conclusions**: We found that lymphocyte and NK cell counts were significantly higher in senior patients with SAD and that NK cell count may be valuable for the prediction of SAD within elderly patient cohorts.

## Introduction

Delirium is a common diagnosis in hospitalized elderly patients (Devlin et al., 2018). As characterized as a mental disorder presenting with global cognitive dysfunction and altered consciousness, sleep cycle, and psychomotor activity (Cole et al., 2009; Devlin et al., 2018), delirium is associated with prolonged intensive care unit (ICU) and hospital stay and increased morbidity (Iwashyna et al., 2010; Sonneville et al., 2017). Among various predisposing and precipitating factors of delirium, infection, which may lead to organ dysfunction or sepsis, is highlighted in the management of delirium of elderly patients in ICU (Eidelman et al., 1996). The signs of neurological involvement including confusion, agitation, and coma occurring during sepsis are summarized as sepsis-associated delirium (SAD), of which the incidence is approximately 50% (Young et al., 1990). Previous studies demonstrated that sustained systemic inflammation may contribute to prolonged or aggravated brain dysfunction (Ely et al., 2004; McGrane et al., 2011; van den Boogaard et al., 2011; Sonneville et al., 2013). Although the impact of increased inflammatory responses and numerous inflammatory biomarkers in SAD diagnosis, prediction, and interventions have been investigated, solid evidence remains insufficient for the clinical aid in the management of SAD (Toft et al., 2019).

In recent years, extensive studies have indicated that the balance between SAD and peripheral immunity is of great importance in the development of neurological damage (Ren et al., 2020). The present study aimed to investigate the role of immune imbalance in elderly patients with SAD using clinical data from combined retrospective and prospective cohorts. We hypothesized that imbalance in the peripheral immunity could be observed from peripheral lymphocyte counts, which are included in standard blood examinations. We then designed a prospective study to compare changes in immunity in senior sepsis patients with or without SAD, along with the predictive value of lymphocyte counts for diagnosis of SAD.

## Materials and Methods

### Study Design

The study consisted of two parts: a retrospective study analyzed the incidence of SAD in critical care settings and its potential association with lymphocyte counts and neutrophil to lymphocyte ratios (NLR) in the peripheral blood, while a prospective trial evaluated quantitative changes in immune status along with their predictive value for early diagnosis of SAD. This study was approved by the institutional review board of Peking Union Medical College Hospital (PUMCH; approval number: JS-1170). Informed consent was obtained from all patients, and the study was registered at chictr.org.cn (identifier ChiCTR-ROC-17010750).

### Retrospective Study

A retrospective analysis of prospectively collected data was carried out on 1,061 consecutive adult patients (aged ≥65 years) diagnosed with sepsis that were admitted to the Department of Critical Care Medicine between May 2013 and Dec 2016. The data collected included complete past medical history; clinical evaluation including records of vital signs and clinical scores; lab tests at admission, 24 and 48 h after ICU admission including complete blood counts and C-reactive protein (CRP). All patients underwent routine blood analysis at admission using a fully automated cell counter in the local hematology laboratory. The NLR was calculated by dividing the absolute neutrophil count by the absolute lymphocyte count from the same sample. We excluded patients who survived less than 24 h in the ICU (25 patients), those with a history of recognition dysfunction (nine patients), and those with neutropenia at ICU admission (16 patients). Follow-up data were extracted from medical records. The primary endpoint was delirium diagnosed within the first 7 days from admission in patients with sepsis.

### Prospective Study

The prospective arm of the study evaluated sepsis patients admitted to the PUMCH between January 2017 and December 2019. Inclusion criteria were: (1) age ≥65 years; (2) ICU stay >24 h; and (3) diagnosis of Sepsis 3.0 (Shankar-Hari et al., 2016). Exclusion criteria were: (1) any condition causing neutropenia; (2) any condition causing primary or acquired immunodeficiency, such as HIV, autoimmune disease at an active stage, hematological disease, or malignant tumors receiving chemotherapy or glucocorticoids within the previous 3 months; (3) life expectancy of <24 h; and (4) failure to meet the inclusion criteria or obtain written consent.

### Delirium Monitoring

We used the simplified Chinese version of the confusion assessment method for the ICU (CAM-ICU) assessment tool to screen all patients for delirium at ICU admission, twice a day (morning and evening) and upon changes or fluctuations in mental status after ICU admission (Wang et al., 2013). In the retrospective study, a detailed review of medical and nursing notes was performed by the investigators for a full evaluation of delirium. CAM-ICU positivity was considered as delirium and the corresponding duration was recorded. Similarly, in the prospective study, delirium was diagnosed by the same method and its onset and duration were the primary outcomes of the study.

### Data Collection

In both the retrospective and prospective studies, patient demographics, clinical data such as mean arterial pressure, heart rate, duration of ventilator treatment, acute physiology and chronic health evaluation (APACHE) II score and sequential organ failure assessment (SOFA) score, and outcomes, such as the duration of ICU and in-hospital stays, ICU and in-hospital mortality, and 28-day mortality were recorded.

**Figure 1:**
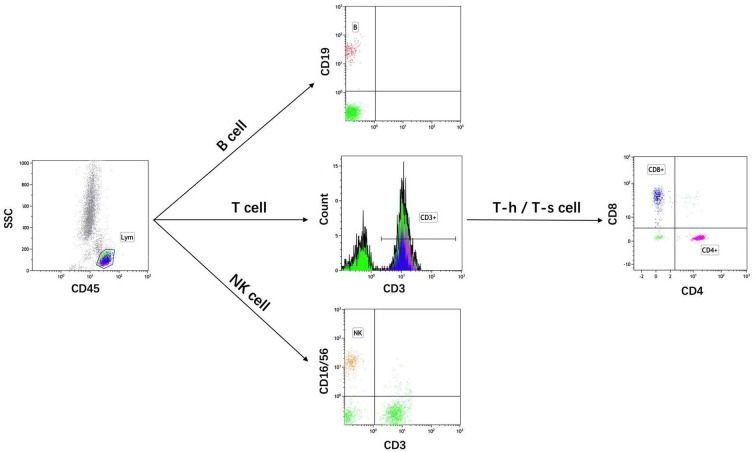
The gating strategy of flow cytometry experiments.

Blood samples were obtained at ICU admission and for a routine examination, including complete blood counts, CRP, procalcitonin, and blood gas analysis. Measurement of immunological parameters was performed on peripheral blood samples in the PUMCH laboratories as previously described (Jalla et al., 2004). In brief, freshly collected EDTA anti-coagulated whole blood was incubated and tested with a panel of monoclonal antibodies labeled with fluorescein isothiocyanate/phycoerythrin/peridinin chlorophyl protein and directed against combinations of CD3/CD8/CD4, CD3/CD16CD56/CD19, and isotype controls (Immunotech, France), then subjected to flow cytometric analysis using a three-color EPICS-XL flow cytometer (Beckman Coulter, Brea, CA, USA) to detect T-cells (CD3^+^), CD4^+^ and CD8^+^ T-cell subgroups, B-cells (CD19^+^), and natural killer (NK) cells (CD3-CD16^+^ CD56^+^). The following fluorescent monoclonal antibodies were used in this study: CD45-FITC/CD4-RD1/CD8-ECD/CD3-PC5, CD45-FITC/CD56-RD1/CD19-ECD/CD3-PC5, and CD16-PE (Beckman Coulter, Brea, CA, USA). The gating strategy of flow cytometry experiments is shown in Figure 1. Rate nephelometry (Array 360; Beckman Coulter, Brea, CA, USA) was used to measure serum levels of immunoglobulin (Ig)A, IgG, and IgM and of complement factors C3 and C4.

### Statistical Analysis

Statistical analysis was conducted using SPSS v19.0 software (IBM SPSS, Armonk, NY, USA). Measurement data were expressed as mean ± SD and compared using independent *t*-tests. Enumeration data were compared using a *χ*^2^ test or with Fisher’s exact test, as appropriate. For detection of correlation, Pearson’s correlation analysis was performed. *P* < 0.05 was considered to indicate a statistically significant difference. Statistically significant variables were subsequently analyzed using a binary logistic regression to identify risk factors associated with the onset of delirium. Only variables markedly associated with SAD (*p* < 0.05) were included in the final model. Receiver operating characteristic (ROC) curves and the area under the curves (AUCs) were examined in significant variables associated with the onset of delirium, to determine a cut-off level and to predict mortality.

## Results

### Retrospective Study

**Figure 2:**
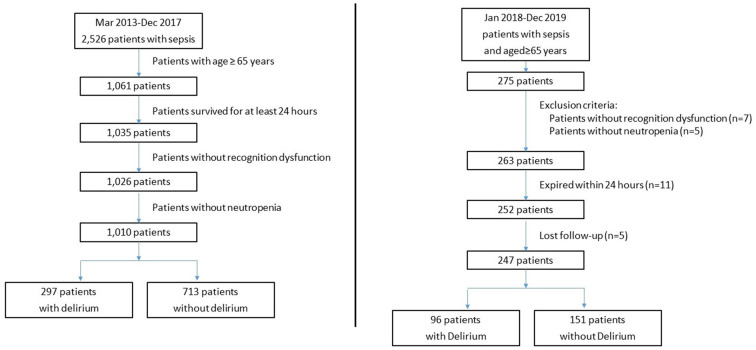
Flowchart comparison of the retrospective and prospective studies.

The number of eligible and excluded sepsis patients is shown in Figure 2. Among 1,010 included adult patients (aged ≥65 years), 297 patients were recorded as CAM-ICU positive and diagnosed with delirium during ICU stay. Patient characteristics of the two groups are provided in Table 1. No significant differences were identified between the groups in age, sex, SOFA score, or lactate at ICU admission (*p* > 0.05), while patients with delirium had higher APACHE II scores. In terms of vital signs, patients with delirium had a significantly higher heart rate and lower temperature and oxygenation index, and a higher proportion received life-sustaining treatments (Table 1).

**Table 1:** Baseline characteristics of the retrospective cohort.

Variables	Delirium (*n* = 297)	Non-delirium (*n* = 713)	*p*-value
Age	74.6 ± 7.1	74.3 ± 7.0	0.315
Sex, male	173 (58.2%)	388 (52.2%)	0.152
APACHE II	20.5 ± 6.6	18.9 ± 7.9	<0.005
SOFA	5.9 ± 4.6	6.0 ± 4.7	0.562
Vital sign			
Heart rate	96.8 ± 20.5	92.5 ± 21.8	<0.005
Temperature	36.5 ± 1.0	36.7 ± 0.9	<0.005
Respiratory rate	18.5 ± 6.8	18.5 ± 7.0	0.944
PFO2	293.3 ± 163.3	341.8 ± 181.4	<0.005
Vital sign			
Lactate	2.4 ± 2.3	2.3 ± 2.7	0.626
Treatment received			
Vasopressor	222 (74.7%)	321 (45.0%)	<0.005
Mechanical ventilation	259 (87.2%)	607 (85.1%)	0.014
Early mobilization	159 (53.5%)	149 (20.1%)	<0.005

*APACHE II, acute physiology, and chronic health evaluation II; SOFA, sequential organ failure assessment*.

Inflammatory parameters, source of infection, and co-morbidities in the two groups of patients are shown in Table 2. The delirium group had a higher proportion of underlying stroke history [56 (18.9%) vs. 92 (12.9%), *p* = 0.007]. No significant difference was identified in the infection source between the two groups, nor any different proportion of other co-morbidities including hypertension, diabetes, or chronic kidney disease. The outcome result is shown in Table 3. Patients with delirium had longer ICU (14.2 ± 14.6 vs. 5.9 ± 10.9 days, *p* < 0.005) and hospitalization times [22.4 (12.1, 40.0) vs. 16.1 (9.8, 27.9) days, *p* < 0.005] and duration of mechanical ventilation (243.0 ± 309.6 vs. 100.1 ± 246.2 h, *p* < 0.005) and vasopressor usage (247.2 ± 304.8 vs. 144.5 ± 274.4 h, *p* < 0.005). However, the 28-day mortality between the two groups showed no statistically significant differences.

**Table 2:** Inflammatory markers, infection source, and co-morbidities of the retrospective cohort.

Variables	Delirium (*n* = 297)	Non-delirium (*n* = 713)	*p*-value
At ICU admission			
PCT level, ng/ml	1.2 (0.29 ± 7.3)	0.88 (0.21 ± 4.29)	0.039
BDG, pg/ml	72.4 (32.2, 142.2)	73.6 (51.3, 137.4)	0.113
CRP, mg/L	117.9 ± 84.4	129.2 ± 79.8	0.574
Neutrophil count (×10^9^/L)	11.3 ± 4.8	10.6 ± 4.2	0.083
Lymphocyte count (×10^9^/L)	1.09 ± 0.32	0.82 ± 0.24	0.001
NLR	13.5 (7.7, 22.0)	17.1 (9.6, 26.4)	0.028
Vital sign			
At 24 h after admission			
Neutrophil count (×10^9^/L)	11.9 ± 3.9	10.9 ± 3.7	0.084
Lymphocyte count (×10^9^/L)	1.06 ± 0.26	0.84 ± 0.18	0.013
NLR	12.7 (8.0, 23.6)	12.8 (7.8, 18.9)	0.318
At 48 h after admission			
Neutrophil count (×10^9^/L)	11.4 ± 3.6	10.9 ± 3.5	0.235
Lymphocyte count (×10^9^/L)	1.07 ± 0.15	0.83 ± 0.19	0.003
NLR	12.5 (7.9 ± 23.7)	14.2 (8.1 ± 24.1)	0.455
Infection source			<0.005
Lung	120 (40.4%)	233 (32.7%)	
Bloodstream	25 (8.4%)	63 (8.8%)	
Abdominal cavity	87 (29.3%)	254 (35.6%)	
Thoracic and mediastinum	9 (3.0%)	15 (2.1%)	
UTI	10 (3.4%)	42 (5.9%)	
Bile duct	11 (3.7%)	44 (6.2%)	
CNS	2 (0.7%)	6 (0.8%)	
Other	13 (4.4%)	26 (3.6%)	
Co-morbidities			
Hypertension	172 (57.9%)	402 (56.4%)	0.324
Stroke history	56 (18.9%)	92 (12.9%)	0.007
Diabetes mellitus	83 (27.9%)	206 (28.9%)	0.495
Chronic kidney disease	21 (7.1%)	49 (6.9%)	0.659

*ICU, intensive care unit; PCT, procalcitonin, BDG, 1, 3-β-D-glucan; NLR, neutrophil to lymphocyte ratio; UTI, urinary tract infection; CNS, central nervous system*.

**Table 3:** Outcomes of the retrospective cohort.

Variables	Delirium (*n* = 297)	Non-delirium (*n* = 713)	*p*-value
ICU stay time, day	14.2 ± 14.6	5.9 ± 10.9	<0.005
Hospitalization time, day	22.4 (12.1, 40.0)	16.1 (9.8, 27.9)	<0.005
28-day mortality	51 (10.9%)	91 (11.1%)	0.458
Duration of mechanical ventilation, hour	243.0 ± 309.6	100.1 ± 246.2	<0.005
Duration of vasopressor usage, hour	247.2 ± 304.8	144.5 ± 274.4	<0.005

**Figure 3:**
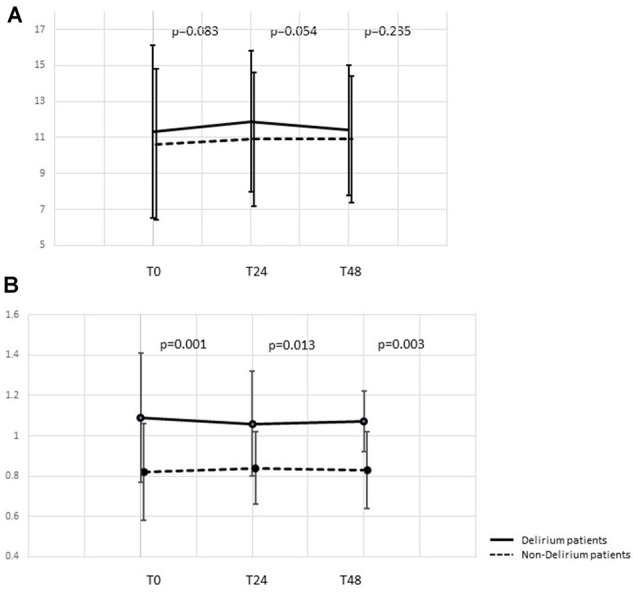
Time course of neutrophil and lymphocyte count dynamic changes.** (A)** Time course of neutrophil counts at 0, 24, and 48 h after ICU admission. **(B)** Time course of lymphocyte counts at 0, 24, and 48 h after ICU admission. ICU, intensive care unit.

Common inflammatory markers and their time courses were also compared. At ICU admission, higher levels of procalcitonin [1.2 (0.29, 7.3) vs. 0.88 (0.21, 4.29) ng/ml, *p* = 0.003], neutrophils (11.3 ± 4.8 vs. 10.6 ± 4.2 × 10^9^/L, *p* = 0.083) and lymphocytes (1.09 ± 0.32 vs. 0.82 ± 0.24 × 10^9^/L, *p* = 0.001) were observed in delirious patients while differences in 1, 3-β-D-glucan (BDG) and CRP were not significant. Comparison at 24 and 48 h after ICU admission showed that the differences in neutrophil counts tended to become non-significant while lymphocyte counts remained statistically significant between the two groups (Table 2, Figure 3). As a derived parameter, the NLR showed similar trends to the neutrophil counts.

Multivariable logistic regression analysis was conducted for all parameters that were statistically different in the univariate analysis. The results indicated that APACHE II score [OR 1.024 (1.008, 1.040), *p* = 0.003], NLR [OR 1.013 (1.003, 1.023), *p* = 0.018], lymphocyte count [OR 1.586 (1.228, 2.047), *p* < 0.005] and history of stroke [OR 1.403 (1.024, 1.922), *p* = 0.035] showed significant associations with delirium (Table 4).

**Table 4:** Multivariable logistic regression analysis on sepsis-associated delirium.

	Unadjusted		Adjusted	
	OR (95% CI)	*p*-value	OR (95% CI)	*p*-value
Age	1.240 (0.988, 1.554)	0.063	1.004 (0.988, 1.021)	0.624
APACHE II	1.028 (1.013, 1.044)	<0.005	1.024 (1.008, 1.040)	0.003
PCT	1.002 (0.997, 1.007)	0.532	1.000 (0.995, 1.006)	0.845
Neutrophil	1.014 (0.997, 1.031)	0.105	1.008 (0.988, 1.028)	0.435
NLR	1.006 (1.000, 1.011)	0.061	1.013 (1.003, 1.023)	0.018
Lymphocyte	1.366 (1.124, 1.660)	0.002	1.586 (1.228, 2.047)	<0.005
Stroke history	1.527 (1.124, 2.075)	0.007	1.403 (1.024, 1.922)	0.035

*APACHE II, acute physiology, and chronic health evaluation II; PCT, procalcitonin; NLR, neutrophil to lymphocyte ratio; CI, confidence interval*.

### Prospective Study

As shown in Figure 2, 275 patients who were over 65 years of age were eligible for enrolment into the study from January 2018 to December 2019 and 247 patients were enrolled. The clinical characteristics of the 247 patients, of which 96 patients were diagnosed with SAD, are shown in Table 5. No significant differences were identified between the two groups in age, sex, APACHE II score, SOFA score, or lactate at ICU admission (*p* > 0.05). The patients who died in 28-day after ICU admission in the prospective cohort were 18 in the non-delirium group and 12 in the delirium group, which correspond to the 28-day mortality of 11.9% and 12.5%, respectively. For inflammatory markers, the patients with delirium had higher levels of procalcitonin [1.1 (0.45, 6.5) vs. 0.81 (0.2, 5.1) ng/ml, *p* = 0.021], lymphocyte counts [0.83 (0.56, 1.15) vs. 0.72 (0.40, 1.06) × 10^9^/L, *p* = 0.020] and lower NLR [13.5 (7.7, 22.0) vs. 17.1 (9.6, 26.4), *p* = 0.028], while the difference in neutrophil counts between the two groups was non-significant.

**Table 5:** Baseline characteristics of the prospective cohort.

Variables	Delirium (*n* = 96)	Non-delirium (*n* = 151)	*p*-value
Age	74.7 ± 6.4	73.8 ± 5.9	0.305
Sex, male	61 (63.5%)	93 (61.6%)	0.758
APACHE II	22.0 ± 7.1	23.1 ± 7.0	0.700
SOFA	5.1 ± 4.6	5.8 ± 4.3	0.622
Lactate	2.6 ± 2.8	2.5 ± 2.3	0.728
At ICU admission			
PCT level, ng/ml	1.1 (0.45, 6.5)	0.81 (0.2, 5.1)	0.021
BDG, pg/ml	82.4 (52.2, 112.2)	91.6 (41.3, 156.2)	0.245
CRP, mg/L	101.2 ± 54.4	135.2 ± 92.8	0.334
Neutrophil count (×10^9^/L)	11.2 ± 7.3	13.0 ± 8.7	0.369
Lymphocyte count (×10^9^/L)	0.83 (0.56, 1.15)	0.72 (0.40, 1.06)	0.020
NLR	13.6 (7.8, 25.9)	15.7 (9.2, 27.0)	0.399

*APACHE II, acute physiology and chronic health evaluation II; SOFA, sequential organ failure assessment; PCT, procalcitonin; BDG, 1,3-β-D-glucan; CRP, C-reactive protein; NLR, neutrophil to lymphocyte ratio*.

**Figure 4:**
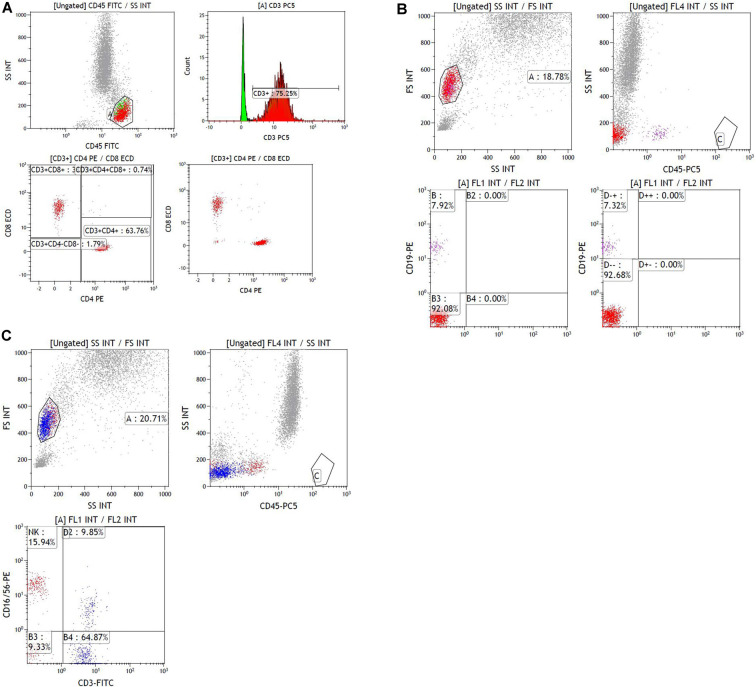
Sample data of flow cytometry experiment.** (A)** Sample data of gating data on T lymphocytes. The gating percentage of [CD3^+^], [CD3^+^CD4^+^] and [CD3^+^ CD8^+^] were 75.25, 47.98 and 25.36%, respectively. **(B)** Sample data of gating data on B lymphocytes. The gating percentage of [CD19^+^] was 7.92%. **(C)** Sample data of gating data on NK lymphocyte. The gating percentage of [CD16^+^CD56^+^] was 15.94%.

**Figure 5:**
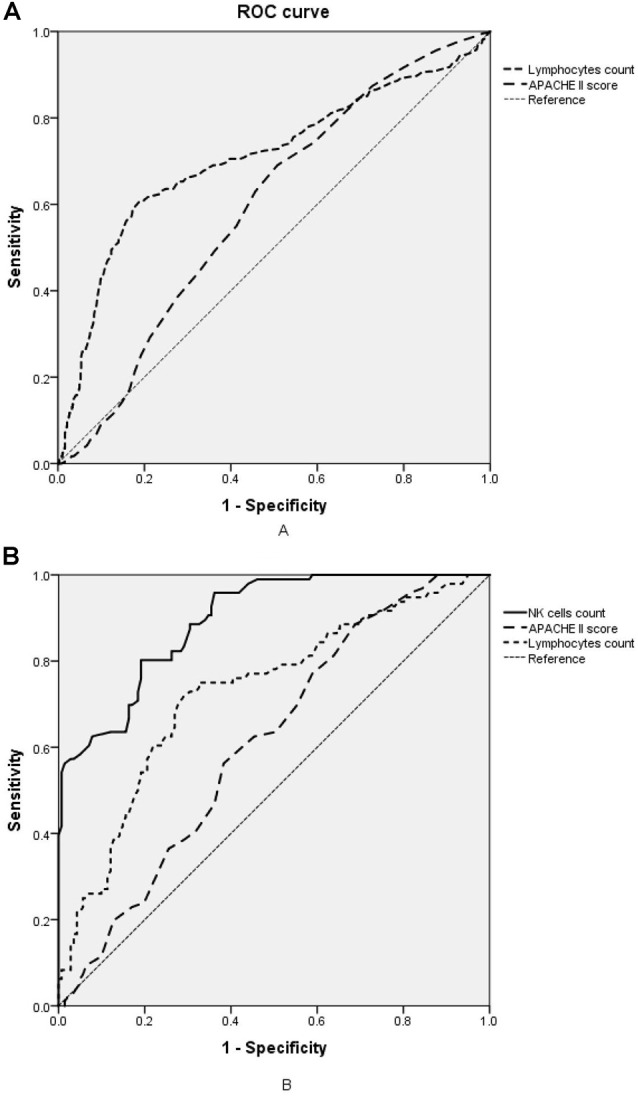
Receiver operating characteristic (ROC) analysis of immune parameters predicting sepsis-associated delirium (SAD).** (A)** ROC curves of APACHE II score and lymphocyte counts in the retrospective cohort. **(B)** ROC curves of NK cell counts, APACHE II score and lymphocyte counts in the prospective cohort. APACHE II, acute physiology and chronic health evaluation II; NK cell, natural killer cell.

The lymphocyte subset results are shown in Table 6 and the demonstration of sample data in the flow cytometry experiment is shown in Figure 4. NK cell counts in the SAD group were significantly higher than those of the non-SAD group [96 (68, 118) vs. 56 (26, 92) cells/μl, *p* = 0.024] while the differences in B lymphocyte, CD4^+^ T lymphocyte, and CD8^+^ T lymphocyte counts between the two groups were not. No significant differences in other studied immune parameters (C3, C4, IgA, IgG, and IgM) were found between SAD and non-SAD patients. We also performed a ROC analysis. Compared with lymphocyte counts and APACHE II score, NK cell counts had the greatest discriminatory ability, with an AUC value of 0.895 (95% CI: 0.857, 0.933), while that of lymphocyte counts was 0.728 (95% CI: 0.662, 0.795) and the APACHE II score was 0.611 (95% CI: 0.540, 0.682; Figure 5). According to the ROC curves, an NK cell count cut-off value of 87 cells/μl in senior septic patients at ICU admission was predictive of a diagnosis of delirium with a sensitivity of 80.2% and specificity of 80.8%, while the cut-off values of APACHE II and lymphocyte count were 21 points and 0.96 × 10^9^/L, respectively. The discriminatory powers of the above three parameters are shown in Table 7.

**Table 6:** Lymphocyte subsets of prospective cohort.

Cell count (cells/μl)	Delirium (*n* = 96)	Non-delirium (*n* = 151)	*p*-value
B lymphocyte	89 (45, 144)	96 (45, 181)	0.109
T lymphocyte	509 (289, 799)	483 (302, 734)	0.118
CD4^+^ T	302 (196, 473)	294 (186, 463)	0.120
CD8^+^ T	148 (75, 301)	135 (72, 236)	0.686
NK cell	96 (68, 118)	56 (26, 92)	0.024
C3, g/L	0.726 ± 0.332	0.781 ± 0.369	0.363
C4, g/L	0.181 ± 0.065	0.167 ± 0.062	0.128
IgA, g/L	2.58 ± 1.23	2.48 ± 1.27	0.602
IgG, g/L	11.2 ± 4.4	10.76 ± 4.9	0.509
IgM, g/L	0.77 ± 0.49	0.77 ± 0.44	0.930

*C3, complement factor 3; C4, complement factor 4; Ig, immunoglobulin; LB, B lymphocyte; LY, lymphocyte; NK cell, natural killer cell*.

**Table 7:** Discriminatory powers of APACHE II, lymphocyte count, and NK cell count in the retrospective and prospective cohorts.

		Area under ROC curve (95% CI)	Sensitivity	Specificity	Predictive positive value	Predictive negative value	Relative risk for delirium (95% CI)
**Retrospective cohort**	APACHE II	0.595 (0.564, 0.626)	38.40%	72.70%	45.40%	72.70%	1.661 (1.605, 2.114)
	Lymphocyte count	0.707 (0.676, 0.739)	60.80%	78.50%	62.20%	79.40%	5.986 (4.671, 7.669)
**Prospective cohort**	APACHE II	0.611 (0.540, 0.682)	56.30%	61.70%	50.00%	67.40%	2.071 (1.223, 3.509)
	Lymphocytes count	0.728 (0.662, 0.795)	71.90%	69.50%	60.00%	79.50%	5.833 (3.319, 10.253)
	NK cells count	0.895 (0.857, 0.933)	80.20%	80.80%	72.60%	86.50%	17.049 (8.945, 32.495)

*The cut-off values of NK cell count, APACHE II score, and lymphocyte count were 87 cells/μl, 21 points, and 0.96 × 10^9^/L, respectively. APACHE II, acute physiology, and chronic health evaluation II; NK cell, natural killer cell; ROC, receiver operating characteristic; CI, confidence interval*.

## Discussion

To our knowledge, this is the first study to show that immune imbalance, as measured by peripheral lymphocyte counts and lymphocyte subsets, is independently associated with SAD in elderly patients in critical care settings. We demonstrated significantly higher lymphocyte counts in patients with SAD, of which increased NK cell count was independently associated with a higher incidence of SAD. This finding supports the role of NK cells and potential neuroinflammation in the development of SAD in senior sepsis patients and provides evidence that evaluation of lymphocyte subtyping is important for early diagnosis and prediction of this pathophysiological process.

In this combined study, a retrospective study was first performed to compare common inflammatory parameters between advanced age patients with and without SAD. Our results showed significantly increased lymphocyte counts in the SAD group during the first 48 h after ICU admission (Figure 3), and multivariable analysis showed that lymphocyte count was independently associated with SAD (Table 4). To further explain this difference, we performed the prospective part of the study, focusing on lymphocyte subsets. Specifically, we found high NK cell counts at ICU admission were associated with a higher incidence of SAD and the ROC analysis confirmed its good predictive performance.

In senior patients, delirium was demonstrated to be associated with a poorer prognosis compared with non-delirious counterparts. In a cohort of ICU patients ≥60 years of age, Pisani et al. (2009) reported a 1-year mortality rate of 40% with a delirium duration of 1–2 days, which rose to 70% if delirium persisted for ≥5 days. As a common complication of sepsis and an independent predictor of death (Ebersoldt et al., 2007), SAD is reported to have an incidence of 70% and its mechanism is incompletely understood, especially in patients of advanced age (Eidelman et al., 1996; Zauner et al., 2002; Simone and Tan, 2011). In this study, our results showed that the SAD was associated with prolonged ICU/hospitalization and ventilation duration while the 28-day mortality between the SAD and non-SAD groups was non-significant. Our results correspond with the inconsistent association between delirium and mortality, especially for 28-day mortality. However, considering the majority of the patients mainly suffered from pulmonary and abdominal infection and the weaning on mechanical ventilation and rehabilitation relied on good compliance and consciousness, it is reasonable that the patients with delirium were associated with significantly prolonged duration of mechanical ventilation and ICU stay time.

Recently, the impact of CNS inflammation has been a key area of investigation in delirium of senior patients (Godbout et al., 2005; Yiru and Xia, 2018; Berger et al., 2019). Although multiple studies have demonstrated the damage from excessive inflammation to the CNS and the association between SAD and “cytokine storm” that manifests as immune factor imbalance (Munford and Pugin, 2001; Oberholzer et al., 2001; Abraham and Singer, 2007), the significance and impact of lymphocyte counts on SAD remain undetermined (Inoue et al., 2015; Egberts and Mattace-Raso, 2017; Kotfis et al., 2019). Our findings support that the lymphocytes, as well as NK cells, may play an important role in the mechanism of SAD, irrespective of the presence or absence of other inflammatory mediators or the influence of inflammation on survival and other organ dysfunctions. Previous studies have demonstrated that NK cells can be swiftly mobilized by danger signals and are among the earliest arrivals at target organs including the inflamed CNS (Shi et al., 2011). Noteworthy, the results of a previous study showed that severe sepsis patients with high levels of NK cells at admission had higher mortality (Andaluz-Ojeda et al., 2011). Hatta et al. (2014) also demonstrated the association between increasing blood NK cell activity and the occurrence of delirium. In the present study, lymphocyte and NK cell counts were significantly higher in SAD patients while other immune parameters including CD4^+^, CD8^+^, and other inflammatory factors were not, suggesting innate immune activation and potential neuroinflammation played an important role in elderly patients with sepsis. According to our results, increased peripheral NK cell counts are an independent predictor of delirium in sepsis patients of advanced age and close monitoring for the occurrence of delirium would be necessary.

Since Macdonald et al. (2007) first reported that high levels of CRP independently predicted the incidence of delirium, attention has been paid to the inflammatory response and the association between delirium and numerous inflammatory biomarkers (McGrane et al., 2011; Toft et al., 2019). However, none of them were demonstrated to effectively assist in diagnosing and predicting delirium. Intriguingly, growing evidence suggests that the non-specific immune system activation may be the initial response during sepsis, leading to an immune imbalance between neutrophils and lymphocytes in the peripheral circulation and acute inflammation including neuroinflammation in delirium among senior patients (Egberts and Mattace-Raso, 2017; Kotfis et al., 2019). Our results show that the levels of peripheral lymphocytes significantly increased in senior patients with delirium and remained elevated during the first 48 h after ICU admission. Hence, as one of the most common and routine tests in clinical practice, complete blood cell tests and differential subset counts should be highly valued in the management of delirium.

This study had several limitations. First, owing to the nature of the retrospective analysis, our study on delirium accepted the most widely used and simple diagnostic criteria of CAM-ICU, instead of the CAM-ICU-7 released in 2017 to improve its performance regarding delirium severity (Khan et al., 2017). Second, our study did not conduct dynamic monitoring of lymphocyte subsets over time to illustrate dynamic changes, which could be improved in future studies. Third, this intervention was performed at a single medical center, and the relatively small sample size might have obscured the detection of some real changes, which may consist of differences in lymphocyte subsets and inflammatory parameters due to a lack of statistical power. Besides, the neuroinflammatory agents such as cerebrospinal fluid analysis or acetylcholinesterase-activity measurement were not investigated in our study since the practical difficulties in clinical. Future work on larger cohorts and multicenter controlled study and deep research on neuroinflammatory reaction is warranted to confirm our conclusions.

## Conclusions

In this combined retrospective and prospective study, we found that the lymphocyte and NK cell counts were significantly higher in senior patients with SAD, compared with age-matched non-SAD patients, and that NK cell counts may be valuable for the prediction of SAD within patient cohorts of advanced age. Our findings highlighted the importance of NK cells and potential neuroinflammation in the progression of SAD in elderly patients and support the addition of lymphocyte subset analysis to the prediction and diagnosis of SAD.

## Data Availability Statement

The raw data supporting the conclusions of this article will be made available by the authors, without undue reservation.

## Ethics Statement

The studies involving human participants were reviewed and approved by institutional review board of Peking Union Medical College Hospital (PUMCH; approval number: JS-1170). The patients/participants provided their written informed consent to participate in this study.

## Author Contributions

DL designed the study and prepared the drafting of this article. NC conceived the study and made final approval of this manuscript. GB and JZ analyzed all data and helped revise this manuscript. WC contributed to the acquisition of laboratory data and JC was in charge of the acquisition of clinical data. All authors contributed to the article and approved the submitted version.

## Conflict of Interest

The authors declare that the research was conducted in the absence of any commercial or financial relationships that could be construed as a potential conflict of interest.
